# Extended prone positioning for intubated ARDS: a review

**DOI:** 10.1186/s13054-023-04526-2

**Published:** 2023-07-05

**Authors:** Thaïs Walter, Jean-Damien Ricard

**Affiliations:** 1 Université Paris Cité, AP-HP, Hôpital Louis Mourier, DMU ESPRIT, Service de Médecine Intensive Réanimation, 92700 Colombes, France; 2 Université Paris Cité, UMR1137 IAME, INSERM, 75018 Paris, France

**Keywords:** Acute respiratory distress syndrome, Prone positioning, Extended prone positioning, Prolonged prone positioning, Mechanical ventilation

## Abstract

During the COVID-19 pandemic, several centers had independently reported extending prone positioning beyond 24 h. Most of these centers reported maintaining patients in prone position until significant clinical improvement was achieved. One center reported extending prone positioning for organizational reasons relying on a predetermined fixed duration. A recent study argued that a clinically driven extension of prone positioning beyond 24 h could be associated with reduced mortality. On a patient level, the main benefit of extending prone positioning beyond 24 h is to maintain a more homogenous distribution of the gas–tissue ratio, thus delaying the increase in overdistention observed when patients are returned to the supine position. On an organizational level, extending prone positioning reduces the workload for both doctors and nurses, which might significantly enhance the quality of care in an epidemic. It might also reduce the incidence of accidental catheter and tracheal tube removal, thereby convincing intensive care units with low incidence of ARDS to prone patients more systematically. The main risk associated with extended prone positioning is an increased incidence of pressure injuries. Up until now, retrospective studies are reassuring, but prospective evaluation is needed.

**Graphical Abstract:**

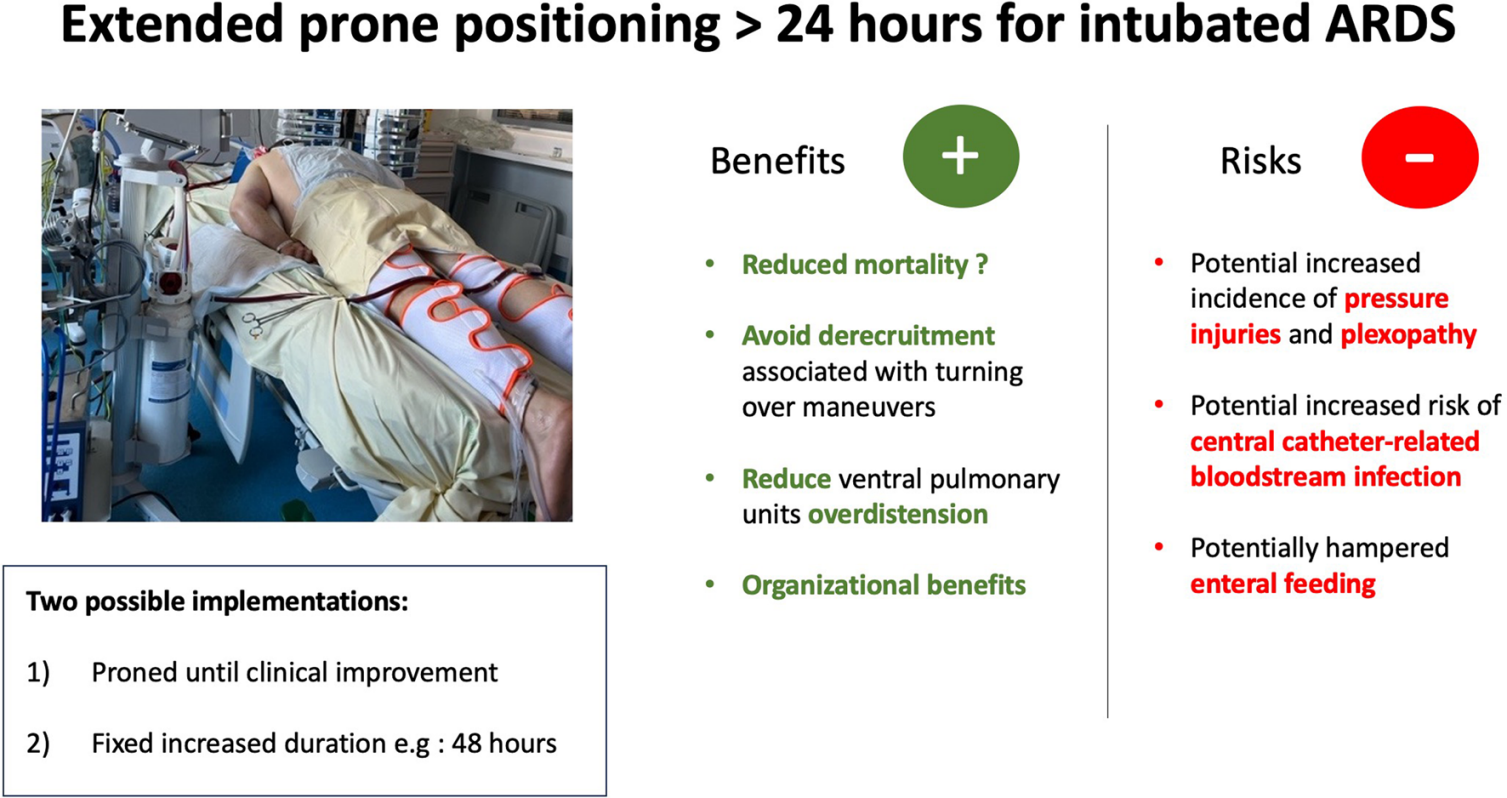

## Background

Prone positioning (PP) is one of the few measures which have demonstrated an impact on patient outcomes with a significant reduction in mortality of mechanically ventilated patients with acute respiratory distress syndrome (ARDS) [1]. It is currently universally recommended for patients with severe ARDS [2, 3]. Failure of early studies to demonstrate a survival benefit of PP in ARDS has been attributed to both the insufficient duration and the late initiation of PP. PROSEVA was the first study to demonstrate a reduction in mortality with a PP duration of 17 h [4]. A meta-analysis further showed that the duration of PP determined the decrease or not of mortality [5]. During the COVID-19 pandemic, with the surge of patients requiring mechanical ventilation and iterative PP sessions [6], sometimes as many as six sessions per patient [7], many more patients were turned prone than before. In this context, several centers have independently reported implementing PP sessions of a duration strictly greater than 24 h [8, 9]. Recently, a retrospective observational study has also provided some arguments that PP of a duration greater than 24 h might be associated with a reduced mortality in COVID-19-related ARDS [10]. In this review, we sought to describe the underpinning rationale of extending PP beyond 24 h and its potential associated complications and to provide a comprehensive summary of the literature reporting the implementation of extended PP, its impact on outcomes, ventilatory parameters and its main associated complications.

## Main text

### Clinical rationale for extending the duration of PP sessions

The main benefit of PP is to homogenize the stress and strain applied by mechanical ventilation on the lungs [11]. The lack of homogeneity in the redistribution of the ventilation volume in the lung has several origins. The first one is a mismatch between the form of the lung and the form of the chest cavity [12]. The lung has approximately the shape of a trapezoid, whereas the chest cavity resembles a cylinder. The negative pleural pressure which allows both shapes to match applies strain on the lung. This strain, however, is not evenly distributed, with the ventral alveolar region being more strained and thus more inflated than the dorsal alveolar regions [11]. The dorsal alveolar units, on the other hand, are subjected to the weight of the overlying lung, which is all the greater with the wet ARDS lungs [13]. This leads to a collapse of the dorsal alveolar units which, in turn, further aggravates the distension of ventral alveolar units. Being turned prone allows both forces, the gravity and the strain, to oppose each other [14].

This more homogenized repartition of ventilation volume, stress and strain is not systematically translated into improved ventilatory parameters. An experimental study on pigs showed that PP induced better homogenized pleural pressure without improving lung compliance [15]. In patients with ARDS, a prospective experimental study on 21 patients showed that PP was associated with the reopening of posterior alveolar units without any improvement in lung static compliance [16]. The main reason is that when patients are returned to PP, the overall compliance of the thoracic cavity decreases because the rigid surface of the bed blocks the ventral side of the thorax. In the absence of recruitment, compliance of patients in PP should decrease and plateau pressure should increase [13]. The stability of the lung compliance after PP is an indicator that further alveolar units have been recruited.

Whether extended prone positioning leads to a more homogenous distribution of the gas–tissue ratio or even only maintains it needs further evaluation by prospective physiological studies. In both cases, extending PP might be beneficial by reducing the time spent in the supine position in the first days after the onset of severe ARDS, thus avoiding the associated pulmonary units overdistention. It might also reduce the number of turning over maneuvers and its associated de-recruitments. The final benefit might be a decrease in systemic inflammation. In a retrospective study where PP was extended up to 72 h, the plasma IL-6 concentration, a marker of systemic inflammation, declined steadily in the prone group, suggesting a close relationship between systemic inflammation and prone position ventilation [17].

### Hemodynamic effects

PP probably has a positive effect on hemodynamics. In the PROSEVA trial, patients randomized to PP had a lower incidence of cardiac arrest [4], and in a meta-analysis, patients receiving PP had a lower incidence of arrhythmias [18]. Overall, PP allows for a decrease in pulmonary vascular resistance, probably due to the reduction in hypocapnia, hypoxemia and plateau pressure [19].

Position change might be associated with hemodynamic compromise [20]. However, a beneficial effect of PP on cardiac index was found in 25% of the sessions, especially in patients with lower cardiac index and lower global ejection fraction before PP [21]. This suggests that hemodynamic instability should not be an obstacle to PP. Using thoracopelvic supports during PP significantly decreases stroke volume (in addition to an increase in contact pressures and no benefit in gas exchange) [22]. Their use should be discouraged. Finally, in patients with right ventricular overload, PP of 18 h was associated with the normalization of right ventricle function and a significant increase in cardiac index [23]. Altogether, this information is reassuring concerning the hemodynamic tolerance in case of further extension of PP duration.

### Organizational benefits

Current guidelines recommend PP to be applied between 12 and 16 h per day, alternating with 8 h of supine positioning [1–3]. Adherence to duration recommendations implies that patients are turned prone between once and twice daily. Such a high frequency has several major drawbacks: intense workload, increased risk of an accidental central venous catheter or tracheal tube removal at each procedure [4, 24] and viral exposure. The high prevalence of overweight and obesity in COVID-19 patients [6] also increases the risks of musculoskeletal injuries for healthcare professionals.

Extending PP sessions over 24 h has two main organizational benefits. The first one is that it significantly decreases the number of PP sessions that have to be performed to get the same total cumulative duration on PP [8, 10, 25]. The second is that it allows switching from a fixed duration to an organizational-oriented duration. In their retrospective study, Walter et al*.* describe how they always (in 94% of PP. sessions) turned patients supine during the daytime, when clinical teams were fully staffed. Had the 16-h duration been strictly applied to this cohort, as in some ICUs [7], more than half of the returns to supine would have occurred during night shift periods, when the medical staffing level is reduced. Because night shifts have been associated with more adverse events such as unplanned extubation [26] and mechanical complications of central venous line insertion [27], concentrating all returns to supine during the daytime might improve the security of the procedure.

More impactfully, this increased duration might also help to convince clinical teams to use PP more extensively. In a retrospective study, Langer et al*.* showed that 25% of patients with severe COVID-19-related ARDS were never turned prone during the course of their stay [6]. A figure which can be as high as 84% in studies preceding the COVID-19 pandemic [28]. In another prospective international one-day prevalence study, only a third of patients with severe ARDS were turned prone. In more than 20% of the cases, the reason for not using PP was that hypoxemia was not severe enough. As this reason directly contradicts international guidelines [1–3], we can hypothesize that other reasons not recognized by the physician came into account when deciding to put patients in PP. One explanation could be the unwillingness of physicians to implement treatment in which the most severe hazards are catheters dislodging (impacting arterial, venous catheters or endotracheal tube and ECMO canula). Reducing the number of turning over session might reduce the risk of catheter dislodging. This might reassure clinicians and help increase adhesion to prone positioning, especially in intensive care stations where ARDS prevalence is low. In this case, however, extra care should be given to avoid pressure injuries. This could take the form of a scheduled check of correct body position, especially in obese and hypoxemic patients.

### Extended PP and complications

The most common complication associated with PP is PI [29]. As COVID-19 patients often require several PP sessions, extending PP sessions might further increase PI cumulative incidence. Walter et al*.* have shown that extending PP to a median of 39 h resulted in a cumulative incidence of PI of grade ≥ II of 26%. This figure is in line with the cumulative incidence of 25% described in the PROSEVA study [4].

Okin et al*.* found a similar result (cumulative incidence of 30%) with proning sessions of a median of 40 h. In this latter study, it should be noted that more than 10% of the sessions had a duration greater than 75 h [8]. Two observational retrospective studies have shown that the cumulative incidence of grade ≥ III PI associated with extended PP remained extremely low (between 0 and 2.5%) [8, 30]. Finally, the occurrence of PI seems to be associated with the cumulated duration of PP and not with the duration of single sessions [8, 31].

The other complication might be regurgitation. Historically, enteral feeding through a nasogastric tube was stopped during PP sessions and resumed when patients were back in the supine position. If this did not lead to nutrition problems when patients were left only 16 h on the prone position, ceasing enteral nutrition when patients are left up to 10 days on the prone position might lead to severe denutrition, especially during a disease with a high level of catabolism such as is the case during COVID-19 [10, 32]. Walter et al*.* have produced reassuring figures, showing that enteral feeding was well tolerated for more than 70% of PP sessions that lasted for a median of 39 h [8].

Central catheter-related bloodstream infections were reported in one study with a cumulative incidence of 5% [9]. Reporting catheter-related bloodstream infections as a cumulative incidence instead of a number of infections per 1000 catheter days renders the comparison with historical cohorts difficult [33]. However, it does seem a bit higher than expected. The main drawback of extended PP is that it limits access to the catheter insertion site, thus preventing its monitoring. This population presented, however, other risk factors, mainly prolonged duration of critical illness, higher body mass index than non-COVID patients with similar disease severity, high frequency of temporary dialysis catheter insertion and administration of immunomodulatory/immunosuppressive therapies including corticosteroids. The question of preventing central catheter-related bloodstream infections during extended PP will require further inquiries.

**Fig. 1:**
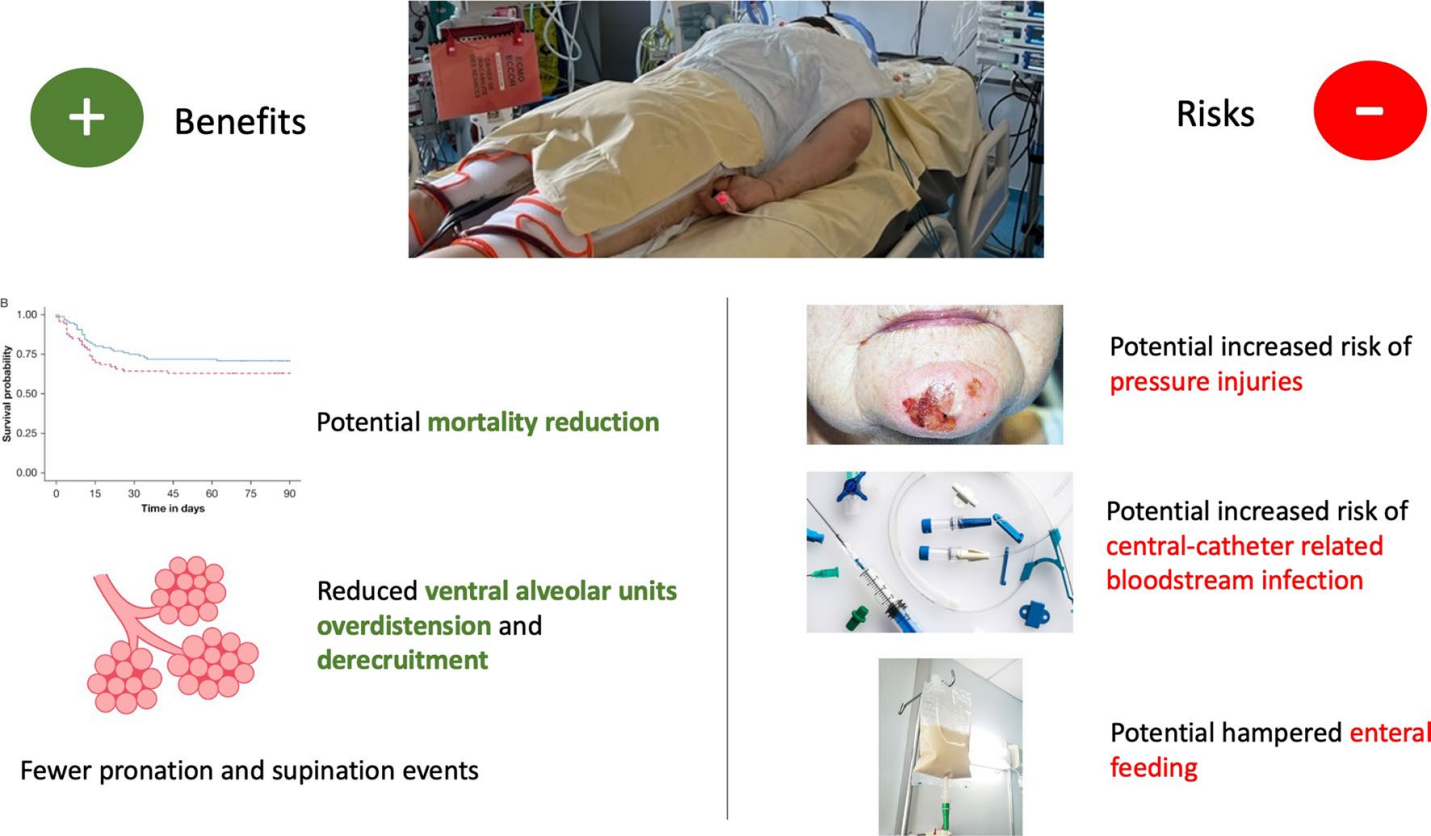
Main benefits and complications associated with prone positioning extended beyond 24 h for intubated ARDS

Long-term complications are mainly plexopathy and more specifically brachial plexus palsy. In a monocentric retrospective study, the incidence of brachial plexus palsy associated with extended PP up to 39 h was lower than the one reported with classical PP duration for COVID-19 patients [8, 34]. Very few studies of high quality are available on the question of whether using the swimmer position does or does not increase the risk of brachial plexus palsy. This question requires further inquiries. Other long-term complications include peripheral nerve injuries, in particular peroneal nerve palsy and injury of the lateral femoris cutaneous nerve and ocular complications [35]. A summary of the potential benefits and complications of extended PP is shown in Fig. 1.

In the prevention of those complications, allied health professionals are key actors. Head nurses are most often responsible for the writing of local protocols for PP installation [36], and knowledge of intensive care unit nurses on PP is probably an important factor in increasing the use of PP [37]. Respiratory therapists might help reduce acute complications such as endotracheal tube removal [38], and dieticians are key actors to optimize enteral feeding tolerance [39]. Rehabilitation physicians and physiotherapists also play an important role in managing long-term complications after ICU discharge [35].

### Review of extended PP sessions before the COVID-19 pandemic

Before the COVID-19 pandemic, five studies described the implementation of PP sessions of a duration longer than 24 h [17, 30, 40–42] (Table 1). In the first study, patients were treated with prone position ventilation for at least 72 h [17]. A significant increase in the P/F ratio was reported between baseline and after 48 h in the prone group. No significant improvement was found in the PaCO_2_ after PP compared to both the baseline and the supine groups. In the second study, patients were turned prone for a mean of 55 ± 7 h [40]. Compared to baseline, patients showed a significant improvement in P/F ratio at the end of the PP session, a significant decrease in PaCO_2_ and the level of plateau pressure. In the third study, patients were turned prone for a median of 47.5 h [30]. A significant increase in the P/F ratio was reported after the first 8 h, with no further significant improvement between the 8th and the 40th hour. Across all three studies, the cumulative incidence of pressure injuries ranged from 13 to 20%, and the number of patients included was very low (< 20 patients).

**Table 1:** Summary of the literature on extended prone positioning for non-COVID-related ARDS

References	Chan et al. [17]	Romero et al. [40]	Lee et al. [41]	Miyamoto et al. [30]	Lee et al. [42]
Etiology	Community-acquired pneumonia	Community-acquired pneumonia	Mostly community-acquired pneumonia	Community-acquired pneumonia and sepsis	Pulmonary and non-pulmonary ARDS
Design	Pseudo-randomized design	Prospective observational	Retrospective	Retrospective	Retrospective
Monocentric or multicentric	Monocentric	Monocentric	Monocentric	Monocentric	Monocentric
Number of patients receiving extended prone positioning	11	15	96	15	116
Country	Taiwan	Chile	Korea	Japan	Taiwan
Effective prone positioning duration in hours (mean, SD or median and IQR)	≥ 72	55 ± 7	78 ± 61	47 [46–67]	66 [44–85]
Criteria for stopping individual prone positioning sessions	At least 72 h and until SaO_2_ ≥ 90% and FiO_2_≥ 60% for 24 h	At least 48 h and until the oxygenation index (FiO_2_* mean airway pressure)/PaO_2_) ≤ 10	PaO_2_/FiO_2_≥ 150 or FiO_2_requirement ≤ 0.5 at PEEP of 8 cm H_2_O or lower, and an improved chest radiography finding or deterioration	Not reported	At least 48 h and until PaO_2_/FiO_2_> 150 mmHg or FiO_2_> 50% with PEEP ≤ 8 cm H_2_O
PEEP level strategy	Set to optimize oxygenation and within authorized combinations of PEEP/FiO_2_such as 14–16 cm of H_2_O for FiO_2_ = 0.9	PEEP titration maneuver, then programmed at 2 cm H_2_O above the point at which the reduction in PEEP generated a fall in the static compliance	≥ 8 cm H_2_O	Not reported	set to optimize oxygenation and within authorized combinations of PEEP/FiO_2_such as 14–16 cm of H_2_O for FiO_2_= 0.9
PEEP before PP in cm of H_2_O (mean, SD or median and IQR)	13 (1)	12 (1)	9.8 (2.6)	13.4 (6.9)	14 [14–16]
Cumulated incidence of pressure injuries	18%	13%	Not reported	20%	Not reported
Grades of the reported pressure injuries	Grade not reported	Grade ≥ II	Not reported	Grade II	Not reported

*COVID* coronavirus disease, *ARDS* acute respiratory distress syndrome, *cm* centimeter, *FiO*_*2*_ fraction of inspired oxygen, *%*: pourcentage, *SD* standard deviation, *IQR* interquartile range, *PaO*_*2*_ partial arterial pressure of oxygen, *PEEP* positive end expiratory pressure, *SaO*_*2*_ capillary saturation in oxygen

In the last two pre-COVID studies, approximately 100 patients were included in each study, and the median PP duration was 78 and 66 h, respectively [41, 42]. However, the evolution of ventilatory parameters between baseline and return to supine position is not reported, nor is the cumulative incidence of pressure injuries.

### Review of extended PP since the COVID-19 pandemic

During the pandemic, and probably because of the number of patients who required PP, ten centers reported their implementation of PP for a duration greater than 24 h for mechanically ventilated COVID-19-related ARDS [8–10, 43–49]. Among them, some studies reported mixed duration of PP sessions with patients treated by both standard and extended duration PP. However, these studies had either very small cohorts [43–45] or did not report specifically on the impact of the extension of PP duration [47].

In the studies presented hereafter, the duration of time prone was extended for all sessions and systematically exceeded 24 h [8–10, 46, 48, 49]. Some studies included 20 patients or less [46, 49]. Among the remaining studies, two strategies were used to extend PP duration. The first is organizational. In a retrospective observational study, a European team reported turning patients prone, whenever possible, during daytime when clinical teams were fully staffed. They were left prone during a period that covered two nights and were returned to the supine position the morning following the second night. If being returned to the supine position was well tolerated, they were left supine for 24 h and then turned to prone position again if the criteria for PP were still met. Otherwise, they were turned back to PP on the same day. It is the only study where PP duration was fixed, i.e., independent of any clinical improvement, similarly as in the PROSEVA protocol. This “two nights” protocol led to a median duration of PP of 39 [IQR 34–42] hours. This protocol allowed for a further improvement of the P/F ratio between H + 16 and just before being returned to the supine position. Moreover, the increase in the P/F ratio during the first PP session was associated with a reduced ICU mortality. No other ventilatory parameters significantly improved between H + 16 and just before being returned to the supine position.

The second strategy for extending PP duration is clinical: Once PP is initiated, it is maintained until clinical improvement. Alternating between PP and supine positioning is completely suppressed. This strategy was first described on a large scale for COVID-19-related ARDS by Douglas et al*.* who published a study that included 427 patients. PP sessions were maintained until patients reached all the following criteria: P/F ratio > 150 with FiO2 > 60% and PEEP levels < 10 cm d’H_2_O [9]. This protocol led to sessions of a median duration of 2.95 days among survivors and 3.3 days among non-survivors with a fourth of session of non-survivors which lasted for at least 6.6 days. Ventilatory parameter evolution was studied over the whole ICU stay and not over single PP sessions.

A Chilian multicentric retrospective study reported the implementation of a national protocol that recommended maintaining PP for at least 48 h and until P/F ratio increased above 200 mmHg. The duration of PP was not associated with a greater reduction in the driving pressure or static compliance between the start of PP and just before being returned to the supine position. This result is coherent with the fact that PP was maintained until clinical improvement.

Finally, a single study evaluated the causal association between clinically driven extended PP duration and mortality [10]. In this multicentric retrospective study, a total of 263 patients were included. Patients were classified in the extended group if the first session lasted > 24 h and in the intermittent group if otherwise. The median PP duration in the extended duration group was 40 h, and the maximum duration of a single session was strictly greater than 10 days. The median PP duration in the standard PP duration group was 17 h. In the study, patients in the prolonged PP group experienced a lower 3-month mortality rate than patients in the standard duration group (adjusted hazard ratio 0.47, 95% CI 0.34–0.67, *P* value < 0.001).

A summary of the different findings of the above-cited study is given in Table 2. Interestingly, none of the studied ventilatory parameters significantly improved in the group of patients who benefited from an extended PP strategy compared with the standard PP strategy. Specifically, no statistical difference was found in the magnitude of change of the ventilatory ratio, the static compliance or in the variation of the P/F ratio.

**Table 2:** Summary of the literature on extended prone positioning for COVID-related ARDS

References	Douglas et al. [9]	Walter et al. [8]	Okin et al. [10]	Cornejo et al. [48]
Etiology	COVID-19	COVID-19	COVID-19	COVID-19
Design	Retrospective	Retrospective	Retrospective	Retrospective
Monocentric or multicentric	Monocentric	Monocentric	Multicentric	Multicentric
Number of patients receiving extended prone positioning	427	81	263	417
Country	United States	France	United States	Chile
Effective prone positioning duration in hours (mean, SD or median and IQR)	2.95 [1.8–5] days among survivors and 3.3 [2.4–6.6] days among non survivors	39 [34–42] h	40 [27–55] h	In patients who required only 1 session (75% of the cohort), the median duration was 4 [3, 4] days
Criteria for stopping individual prone positioning sessions	FiO_2_ < 60% and PEEP levels < 10 cm d’H_2_O during > 4 h	Fixed duration: PP maintained over 2 nigths	To the discretion of the treating physician	At least 48 h and until P/F ≥ 200 mmHg
Adjunctive therapy in case of COVID-19 related ARDS	Not specified	Dexamethasone and Tocilizumab	Remdesivir and Tocilizumab	Not specified
PEEP level strategy	Based on ARDSnet “high” PEEP table	Minimum of 8 cm of H_2_O with a plateau pressure ≤ to 30 cm H_2_O	Set at best compliance or based on ARDSNet PEEP/FiO_2_table	No specific PEEP titration strategy
PEEP before PP in cm of H_2_O (mean, SD or median and IQR)	14 [12–18]	12 [10–13]	12 [10–14]	10 [8–12]
Cumulated incidence of pressure injuries	72%	25%	29%	36%
Grades of the reported pressure injuries	Grade ≥ I	Grade ≥ II	Not reported	Grade I & II

*COVID* coronavirus disease, *ARDS* acute respiratory distress syndrome, *cm* centimeter, *FiO*_*2*_ fraction of inspired oxygen, *%* pourcentage, *SD* standard deviation, *IQR* interquartile range, *PaO*_*2*_ partial arterial pressure of oxygen, *PEEP* positive end expiratory pressure, *SaO*_*2*_ capillary saturation in oxygen

### Advice and recommendation for future studies on extending PP duration

With Okin et al*.* study [10], more studies on extended PP duration are probably to come. We thought it could be interesting to summarize and standardize all the information awaited from a study which would inquire into the impact of PP duration on ARDS outcome.

Firstly, although ARDS physiopathology seems to be not that different between COVID-19 and non-COVID-19-related ARDS [50], mixing both etiologies in a study might require some additional reflection. Indeed non-COVID-19-related ARDS is associated in more than 75% of cases with sepsis [28] which in turn might cause cutaneous perfusion alterations [51] and be a risk factor for PI. Whatever the chosen protocol, it is extremely important to report on both the exact protocol that allows for the implementation of these extended duration and to show the actual distribution of these PP sessions’ duration using an empirical cumulative distribution function. The duration during which patients are left supine between two PP sessions has probably a high clinical impact too and should thus be reported. Concerning PP complications, the cumulative incidence of PI of grade ≥ II should be reported, as well as the incidence of brachial injury, which should be investigated once the patient is back in a conventional ward or a rehabilitation center. Finally, the percentage of sessions during which patients could be enterally fed should also be reported.

## Conclusion

Extending PP duration for more than 24 h is probably feasible and safe with a cumulative incidence of PI of grade ≥ II like the one associated with PP for a duration of between 16 and 24 h. Two strategies have been reported in extending PP duration: one applied a fixed duration, around 40 h [8], and the other maintained patients in prone position until they reached the clinical criteria when PP was no longer indicated [9, 10]. This extended duration has one organizational advantage, as it allows for the reduction in the number of sessions performed and for returning to supine position during the daytime. Furthermore, one retrospective study showed that extending PP duration to 40 h might be associated with reduced mortality. Further prospective, interventional studies are required to confirm these preliminary results.

## Data Availability

Not applicable.

## Abbreviations

ARDS: Acute respiratory distress syndrome
CI: Confidence interval
COVID-19: Coronavirus disease 2019
ECMO: Extracorporeal membrane oxygenation
ICU: Intensive care units
IQR: Interquartile range
PEEP: Positive end expiratory pressure
P/F: Partial pressure of arterial oxygen/fraction of inspired oxygen
PI: Pressure injury
PP: Prone positioning
